# A Laboratory-Scale Evaluation of Smart Pebble Sensors Embedded in Geomaterials

**DOI:** 10.3390/s24092733

**Published:** 2024-04-25

**Authors:** Syed Faizan Husain, Mohammad Shoaib Abbas, Han Wang, Issam I. A. Qamhia, Erol Tutumluer, John Wallace, Matthew Hammond

**Affiliations:** 1 Department of Civil & Environmental Engineering, University of Illinois Urbana-Champaign, Urbana, IL 61801, USA; mabbas22@illinois.edu (M.S.A.); hanwang8@illinois.edu (H.W.); qamhia2@illinois.edu (I.I.A.Q.); tutumlue@illinois.edu (E.T.); 2 Tensar International Corporation, Alpharetta, GA 30009, USA; jwallace@tensarcorp.com (J.W.); mhammond@tensarcorp.com (M.H.)

**Keywords:** aggregate, sensor, resilient deformation, geogrid, triaxial test

## Abstract

This paper introduces a novel approach to measure deformations in geomaterials using the recently developed ‘Smart Pebble’ sensors. Smart Pebbles were included in triaxial test specimens of unbound aggregates stabilized with geogrids. The sensors are equipped with an aggregate particle/position tracking algorithm that can manage uncertainty arising due to signal noise and random walk effects. Two Smart Pebbles were placed in each test specimen, one at specimen’s mid-height, where a geogrid was installed in the mechanically stabilized specimen, and one towards the top of the specimen. Even with simple raw data processing, the trends on linear vertical acceleration indicated the ability of Smart Pebbles to assess the geomaterial configuration and applied stress states. Employing a Kalman filter-based algorithm, the Smart Pebble position coordinates were tracked during testing. The specimen’s resilient deformations were simultaneously recorded. bender element shear wave transducer pairs were also installed on the specimens to further validate the Smart Pebble small-strain responses. The results indicate a close agreement between the BE sensors and Smart Pebbles estimates towards local stiffness enhancement quantification in the geogrid specimen. The study findings confirm the viability of using the Smart Pebbles in describing the resilient behavior of an aggregate material under repeated loading.

## 1. Introduction

In the field of transportation geotechnics, it is important to study the behavior of constructed aggregate layers subjected to vehicular traffic loading. The magnitude and the dynamic nature of such repeated loading lead to loss of pavement structural integrity and permanent deformation accumulation in road foundation layers, i.e., the unbound aggregate base/subbase and subgrade [1]. Ensuring the stability of the unbound aggregate base (UAB) layer under repeated loading, therefore, becomes one of the primary objectives for engineering design and construction. For instances where the layer stability cannot be achieved with compaction alone, stabilization becomes necessary. Stabilization typically refers to improving physical properties and engineering behavior of geomaterials through chemical or mechanical interventions. Mechanical stabilization using geosynthetics, such as geogrids and geotextiles, has become increasingly popular owing to its constructability and rapid implementation. Geogrids, in particular, are known to provide lateral restraint and local stiffness enhancements through aggregate–geogrid interlock. Depending on the manufacturing process, geogrid types used in pavement base layer stabilization can be extruded with various geometries, woven, and welded [2]. A considerable effort in research has been focused primarily on quantifying geogrid benefits through numerical modeling [3,4], the use of embedded sensors such as the bender element (BE) sensors [5,6], and in situ evaluations [7].

Previous research efforts have focused on developing an understanding of geogrid stabilization mechanisms and optimal geogrid location in granular bases [8,9,10,11]. Haas et al. [8] reported on the measurable response improvement of geogrids when placed in the base layer of a paved road. Their research findings provided recommendations on the placement locations of geogrids in a base layer. For a thicker base (>10 in. or 250 mm), the optimal location for a geogrid was determined to be the mid-depth, whereas for a thinner base layer, the optimal location was suggested to be the subgrade and base interface [8]. The effectiveness of geogrid installation at the subgrade and base interface has since been confirmed in other studies for thin base layers or weak subgrade [7,9,10]. However, in the study by Moghaddas-Nejad and Small [10], there was no measurable difference between the performance of geogrid installed at the bottom or in the middle of the base layer under non-channelized traffic. In the study by Al-Qadi et al., the researchers argue that placing a geogrid in the upper one third of a thick (>10 in. or 250 mm) base layer yielded an optimal performance [7]. Moreover, the Federal Highway Administration (FHWA) published guidelines on design and construction practices pertaining to the use of geogrids and other geosynthetics [12]. The manual provides sufficient evidentiary insight on geogrid selection, design, and construction. Moreover, recent research studies [5,13,14,15,16,17,18,19] have considered directly measuring the influence of geogrids on aggregate movement under repeated loading. Notably these studies have focused primarily on railroad ballast, with a few studies focusing on aggregate movement in pavements [17,18,19].

Liu et al. [13,14] first presented the idea of using “SmartRock” for monitoring ballast particle movements. SmartRock is a 3D-printed shell that houses an inertial measurement unit (IMU) sensor. The 3D-printed shell, which is manufactured to a ballast-sized rock-like shape, allows less disturbance and a better simulation of the mechanical response of a ballast aggregate to repeated loading. The researchers performed simulations using the discrete element method (DEM) and observed the important effects of ballast shape properties on repeated load response. The accelerations measured by SmartRock helped determine the primary factors affecting the permanent deformation accumulation in a ballast layer related to lateral accelerations and rotations measured by SmartRocks. In follow up studies by Liu et al. [15,20], the researchers used SmartRocks to evaluate the effect of sensor placement depth and geogrid stabilization on particle movements. They concluded that the particle movement was reduced with depth in a ballast layer. Moreover, the particle movements and rotations diminished near the geogrid. In another study, Liu et al. [16] used SmartRocks to delineate the ballast particle movements in track locations with soft subgrades or mud spots under train traffic. All the previous research efforts demonstrate that an inertial measurement unit (IMU) sensor, when implemented to appropriately simulate aggregate particle morphology, can measure the influence of repeated loading on aggregate movements.

Attempts have also been made towards applying smart sensing technology in the field of pavement geotechnics. Initial results on compaction monitoring in pavement base layer were reported by Wang et al. [17]. The study showcased the ability of SmartRocks to measure changes in the compactive effort and corresponding changes in layer characteristics. Additionally, data trends suggested that controlling particle rotations could help enhance compaction effectiveness of an unbound aggregate base layer. Notably, geogrids provide such rotational restraint through geogrid-aggregate interlocking. Wang et al. [17] recommended future efforts should focus on establishing relationships between particle movement and base layer material properties. In summary, the current state of the art research has focused primarily on and successfully assessed the aggregate movement using linear and rotational acceleration data. However, there exists a strong motivation for estimating the elastic recoverable or resilient deformations experienced by the aggregate particles under repeated loading. This paper, therefore, discusses the development and application of the newly developed Smart Pebble sensor. The study aims to explore the potential applications of this smart sensing technology and report on the preliminary results of Smart Pebble-quantified resilient movements experienced by an aggregate particle under repeated loading in controlled, repeatable laboratory test conditions.

## 2. Objective and Scope 

The primary objective of this paper is to introduce Smart Pebble sensor technology for assessing aggregate movements in an unbound base layer under various stress states during a repeated load triaxial test. For this, two Smart Pebbles were placed at different heights in two triaxial specimens. While the first control triaxial specimen was prepared using only a dense-graded pavement base coarse aggregate material, the second specimen was prepared using a biaxial geogrid placed at 6 in. (152 mm) from the top or mid-height of the specimen using the same aggregate material engineered to the same gradation. The specimens were also instrumented with bender element (BE) shear wave transducer pairs for simultaneously tracking the shear wave velocities. The preliminary acceleration and rotation responses of Smart Pebbles obtained from the specimen top and mid-height locations are discussed. Finally, the resilient deformations estimated for the two Smart Pebbles using a newly developed sensor position tracking algorithm are presented.

## 3. Laboratory Test Equipment

### 3.1. Smart Pebbles

**Figure 1:**
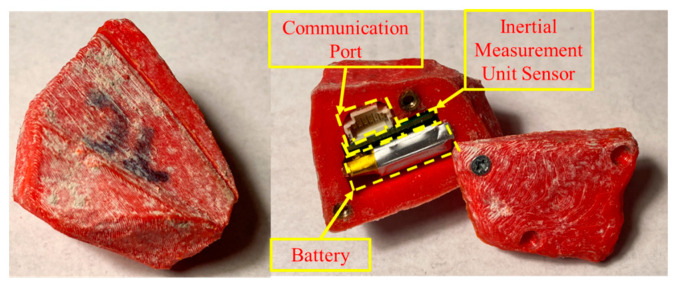
The 3D-printed casing and internal components of a Smart Pebble.

Smart Pebbles are proprietary IMU systems under development by researchers at the Tensar^®^ Corporation in Alpharetta, GA, USA. These sensors can be used to track the linear and rotational translation of aggregates in an unbound granular layer of a pavement. Currently, Smart Pebbles are capable of evaluating aggregate materials in a laboratory setup where the data could be extracted after testing using a wired communication port. The outer shell of the sensor is a 3D-printed casing with custom insert housing for a power source (rechargeable battery), IMU board, and communication port, as shown in Figure 1. The sensors are capable of pre-programmed data collection at variable data acquisition frequencies. The IMU board installed in the sensor is programmed to record the three-axis linear acceleration and gyroscopic rotation. The data acquisition can be programmed to start and stop by the user, and data can be filtered for both low and high frequency noises. Up to 20 Smart Pebbles can be programmed and queried for data simultaneously using a data acquisition box. The measurement data are saved as a comma-separated value (.CSV) file on board a flash memory unit. The collected data are time stamped and synchronized to the parent computer clock, which is used to program the data acquisition scheme. Smart Pebbles offer several advantages for monitoring pavement performance trends through individual aggregate particle and layer assembly responses studied. Their ability to capture detailed particle movement data can provide critical insights into aggregate behavior under dynamic traffic loads, essential for pavement engineering applications. The ability to custom design the external casing with 3D printing allows for matching natural aggregate morphology, preventing any inclusion effects on the sensor measurements. Although wired communication and physical retrieval requirements limit the sensor measurements to laboratory scale, current and ongoing development of Smart Pebbles with wireless capabilities can resolve this limitation. 

### 3.2. Position Tracking Algorithm

**Figure 2:**
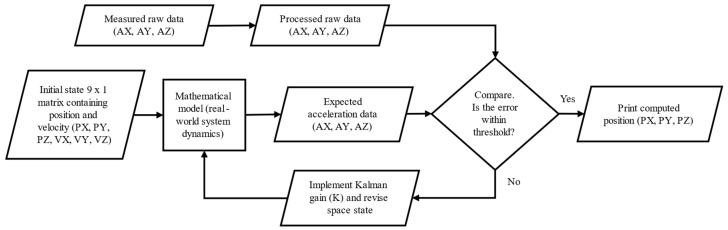
Data processing and position tracking algorithm.

It is often difficult to obtain reliable displacements from an IMU sensor due to inherent noise and resulting sensor drift when double integrated. The primary engineering challenge is to suppress direct current (DC) drift and random walk effects. More information on these challenges are beyond the scope of the study and are presented elsewhere [21]. For the present work, a Kalman filter-based algorithm [22], accompanied by a mathematical model to efficiently estimate the position of the sensor in space (referred to hereafter as “algorithm”), was developed to overcome such issues using a series of scaling, filtering, and feedback reiteration to compute resilient displacements. A procedural overview of the algorithm is provided in Figure 2. 

First, the mean acceleration is shifted to zero to remove random walk effects. This is achieved simply by subtracting the mean acceleration value from the entire raw data. Then, the data are scaled down based on sensor voltage to measurement unit conversion. Additionally, due to random noise variations associated with data acquisition frequency, the scaling factor is also treated as one of the tuning parameters for this algorithm. The Kalman filter algorithm can then be implemented. The Kalman filter works iteratively to predict the state at the next time step and then updates this prediction based on the new measurement. The feedback is based on a mathematical model of an idealized system and assumes the presence of an inherent noise. The latter is true for IMU sensors due to both DC drift and random walk effects.

The Kalman filter in this application was designed to model the motion of an object in the three x, y, and z axes based on accelerometer readings. More specifically, the implemented Kalman filter includes a nine-dimensional state vector, encompassing the position, velocity, and the acceleration in the x, y, and z directions. The state transition matrix, based on Newton’s second law of motion, defines the mathematical estimate of the state at the next time step based on previous time step. On the other hand, the measurement matrix, a critical element of the measurement model, maps these state variables to the measured data, in this case, the accelerometer readings from the Smart Pebbles. 

To account for the system’s inherent noise, noise covariance matrices are introduced, representing the uncertainty linked with the measurements and the process. The configuration of the measurement noise covariance matrix is influenced by the accelerometer data’s variance in each axis, reflecting the noise within the readings. The process noise covariance matrix, symbolizing the uncertainty in the mathematical model’s predictions, is created using a discrete white noise model, capturing the process uncertainties. With the filter applied to the accelerometer readings, every new measurement leads to an update in the state estimate. This adaptive process persistently fine tunes the displacement calculations as it iterates through the next step. It should be noted here that for the purposes of this study, and in line with standard practice of resilient modulus computation, only the last five cycles from each stress stage were considered for computing the resilient deformations. The application of the Kalman filter therefore offers a new method for determining displacement from noisy accelerometer data, effectively bypassing the constraints of simpler integration techniques. By recognizing and adjusting for the inherent uncertainty in the measurement process and using recursive estimation’s abilities, the proposed algorithm facilitates more precise and reliable displacement computations. This capability bears substantial future potential for improving the accuracy of tracking aggregate particle movements.

### 3.3. Repeated Load Triaxial Test Setup (TX-12)

The repeated load triaxial tests were performed using the TX-12 setup at the University of Illinois Urbana-Champaign. The setup is designed to accommodate a cylindrical specimen measuring 12 in. (305 mm) in height and 6 in. (152 mm) in diameter. The setup is connected to a hydraulic pump capable of generating deviatoric stresses corresponding to the AASHTO resilient modulus test procedure (T 307) [23]. The hydraulic pump is connected to an actuator which imparts the deviatoric stresses on the specimen. The deviatoric stress is recorded using a load cell mounted on a steel plate on top of the specimen, which provides a uniform surface for loading the specimen. The top plate also houses two linear variable differential transformer (LVDT) probes mounted at 180° from each other. These LVDTs measure both resilient (or recoverable) and permanent deformations across the height of the specimen. A pneumatic pump provides a continuous supply of pressurized air which is controlled using an analog pressure gauge valve. The accuracy of confinement is ensured using a digital pressure gauge attached to the test chamber. 

**Figure 3:**
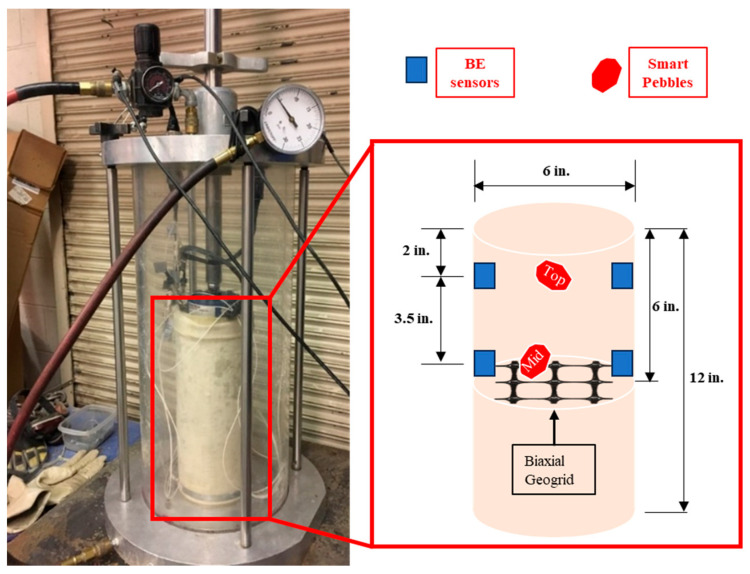
Laboratory setup for the TX-12 device and details on sensor placement in the geogrid specimen (1 in. = 25.4 mm).

For the study, two sensors (namely Top and Mid) were placed at 2 in. (51 mm) and 5.5 in. (140 mm) from the top of the specimen, respectively. The purpose of the Top sensor was to provide calibration reference for the Mid sensor during the development of the position tracking algorithm. The calibration was performed by ensuring that the resilient deformations computed using the Top sensor data approach the resilient deformations measured using the LVDT probes, which serves as the ground truth for bulk specimen deformations. The optimal data scaling parameter obtained during calibration is then applied on the data for the Mid sensor to compute the resilient deformations at the specimen mid-height. Details on the laboratory test setup, Smart Pebble, and BE sensor placement in the specimen are provided in Figure 3 and Table 1.

Note that a similar sensor placement strategy was used for the control specimen. The sampling frequency was varied across the testing scheme to assess the sensitivity of the developed position algorithm to changes in data acquisition rate. The specimens were pulsed using a modified test sequence adopted from the AASHTO T 307 procedure. The applied stress states are presented in Table 2. The deviatoric stresses were applied in haversine shaped load pulses with 0.1 s loading and 0.9 s rest periods. The resilient modulus ($M_{R}$) is computed using the following Equation (1).

(1) $$M_{R}=\frac{\sigma_{d}}{\varepsilon_{r}}$$

where $\sigma_{d}$ is the deviatoric wheel load stress pulsed and $\varepsilon_{r}$ is the resilient (or recoverable) vertical strain. As evident from Equation (1), the specimen material response characteristic that controls the resilient modulus is the resilient strain. Resilient strain is computed as the ratio of the elastic recoverable or resilient deformations to the original height of the specimen. However, direct improvements in resilient modulus as a result of geogrid placement, even at laboratory scale, are difficult to ascertain [6]. This is because geogrid influence is rather localized and may not be pronounced in bulk on-specimen measurements. To this end, recent research efforts in BE shear wave technology have proven effective on laboratory as well as full scale [6]. In the present research, the localized Smart Pebble measurements paired with BE sensor measurements are designed to provide a unique insight into the near geogrid lateral restraint mechanism and its effectiveness in restricting movements of aggregate particles.

### 3.4. Bender Element Sensors

Two pairs of BE sensors were used to measure the shear wave velocity ($V_{s}$) of in horizontal direction across each aggregate specimen. Each BE sensor is a layer of conductive metal sandwiched between two piezoelectric plates. These plates are then subjected to alternating voltage differences, which lead to a controlled cyclical bending strain development in the BE sensor, similar to the action of a fish tail. When embedded in granular media, this movement produces an elastic shear wave. By placing another BE sensor on the opposite end of the specimen, the elastic wave signature is received and visualized through an oscilloscope. The first arrival time can then be determined from the time difference between excitation and reception. The $V_{s}$ is simply computed as the ratio of the tip-to-tip distance between the two BE sensors and the first arrival time. The small-strain shear modulus ($G_{max}$) and the corresponding small-strain elastic modulus ($E_{BE}$) can then be calculated as important material properties using Equations (2) and (3) as follows:

(2) $$G_{max}=\rho V_{S}^{2}$$

(3) $$E_{BE}=2G(1+\nu )$$

where ρ and ν are aggregate material’s bulk density and Poisson’s ratio, respectively. More information on BE sensors and the data interpretation can be found elsewhere [6,24]. In this study, BE sensors were strategically installed at the same depths as the Smart Pebble sensors (see Figure 2). This allowed the researchers to relate the shear wave velocity trends with the resilient deformation trends. Note that resilient modulus (*M_R_*) relates the materials overall resilient response to repeated loading, whereas the small-strain shear modulus measured by the BE sensor pairs primarily capture the local stiffening effect of the geogrid.

## 4. Materials

### 4.1. Aggregate Properties

**Figure 4:**
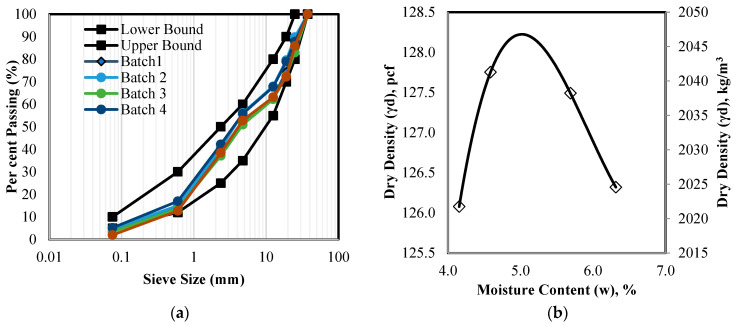
Aggregate properties (**a**) particle size distribution and INDOT No. 53 gradation band and (**b**) moisture–density curve per ASTM D698.

Crushed limestone aggregates conforming to INDOT No. 53 gradation were used in this study. Dry sieving was carried out on four representative batches to develop as-received particle size distribution curves per **ASTM C136** [25] procedure. The results of the dry sieving are shown in Figure 4a. The material had an average particle size ($D_{50}$) of 0.16 in. (4 mm) with a top particle size of 1 in. (25 mm). The moisture–density characteristic curve, established as per the standard Proctor **ASTM D698** [26] test procedure, is presented in Figure 4b. The material exhibited an optimum moisture content (OMC) of 5.1% and a maximum dry density of 128.2 pcf (2060 kg/m^3^). The control specimen was compacted to a bulk density of 133.4 pcf (2137.5 kg/m^3^) while the geogrid specimen was compacted to a bulk density of 134.1 pcf (2148.5 kg/m^3^), both at OMC.

### 4.2. Geogrid Type and Properties

**Figure 5:**
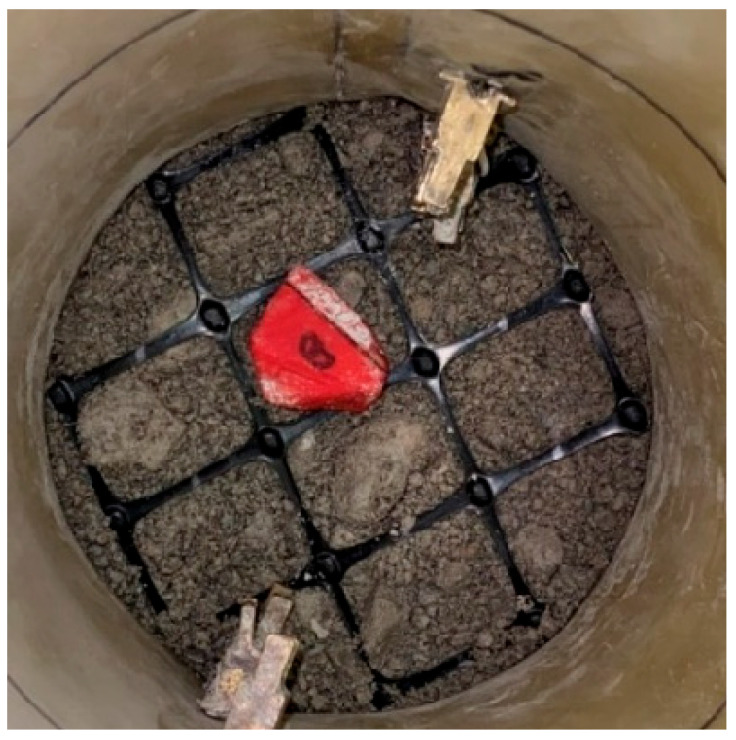
Geogrid and Smart Pebble placed at the TX-12 specimen’s mid-height.

A biaxial extruded geogrid with a square aperture as shown in Figure 5 was used in the study. The dimensions and pertinent characteristics of the geogrid are presented in Table 3. Based on certain recommendations from previous research [27], the ratio of aperture size to average particle size (D_50_) should be more than 2.5 for developing strong interaction between the geogrid and aggregate. Therefore, with a D_50_ value of 0.16 in. (4 mm), any geogrid with an aperture size greater than 0.4 in. (10 mm) will be sufficient to develop appropriate aggregate–geogrid interlock. In accordance, the selected geogrid with a 1.3 in. (33 mm) square aperture size was deemed appropriate in this study.

## 5. Results and Discussion

### 5.1. Vertical Acceleration

**Figure 6:**
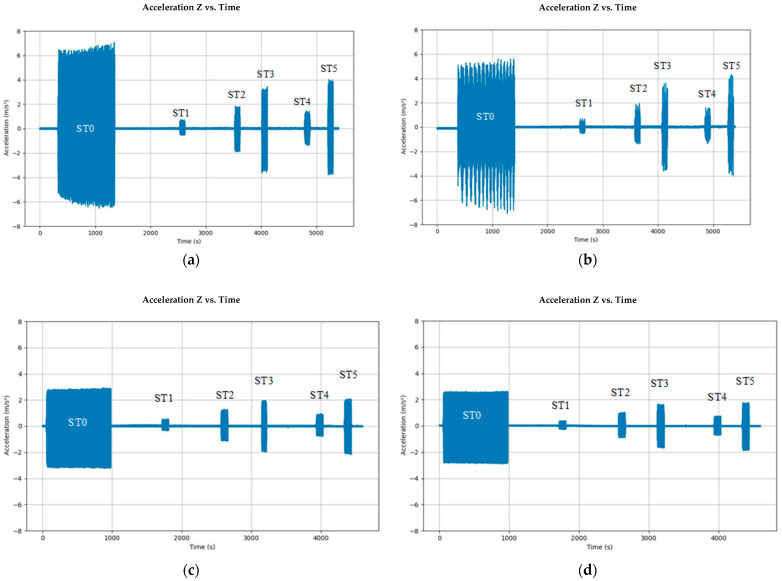
Processed vertical acceleration data from Smart Pebbles: (**a**)Top-CO, (**b**) Mid-CO, (**c**) Top-GG, and (**d**) Mid-GG sensors.

Figure 6a–d present the overall linear vertical acceleration trends from the four Smart Pebble sensors installed in the control and geogrid specimens. Linear vertical acceleration pertains to the sensor’s acceleration data measured in the direction opposing gravity, captured, and depicted over time. The recorded data are plotted against time. The magnitude of the cyclic linear acceleration is clearly driven by the repeated loading experienced by the sensors. Note that, even under zero loading and after filtering, there exists some inherent sensor noise. This further necessitates the particle tracking algorithm and optimizing scaling parameters using the LVDT data as the ground truth. Additionally, a measurement anomaly was observed in the Top-GG sensor data at stress state ST3. Note that this anomaly occurred in between the loading stages ST2 and ST3. Therefore, it will not affect the resilient deformation computations, which are performed using the last five cycles only for each stress state. Therefore, the anomalous data were replaced with background sensor noise and the final plot is shown in Figure 6c.

As evident from the acceleration magnitudes for both control and geogrid specimens, the acceleration measured by the sensor at the specimen’s mid-height is smaller compared to that observed at the top of the specimen. The same trend persists over all the stress states: ST0 (conditioning) through ST5. The highest acceleration trends were observed consistently for the conditioning stage. For the same stress ratio (deviator stress divided by confining pressure, i.e., ST0, ST1, and ST4), the deformation and in turn the acceleration always increase as the deviator stress increases. On the other hand, with the same confining pressure (i.e., ST1, 2, and 3; ST 4 and 5), the deformation and, in turn, the acceleration stay similar or increase as the deviator stress increases. The higher deviator stress and deformation will still lead to a stress-hardening behavior of unbound aggregates as demonstrated through resilient modulus trends observed for the ratio between the two.

### 5.2. Resilient Deformation

**Figure 7:**
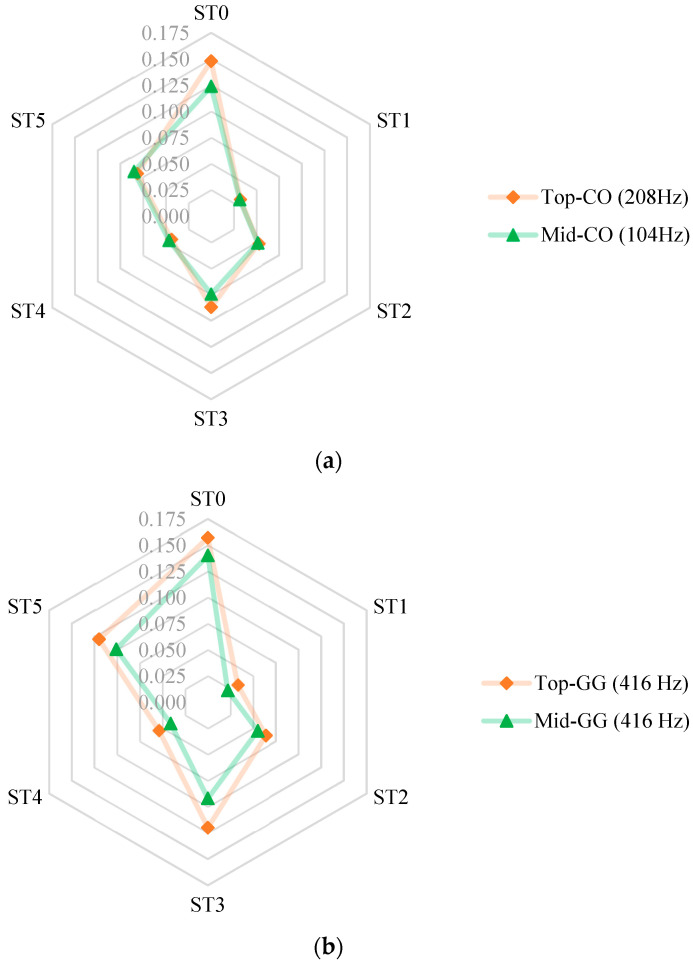
Comparing resilient deformations recorded by the two Smart Pebble sensors installed in (**a**) the control specimen and (**b**) the geogrid stabilized specimen. Notes: The vertical axis shows resilient deformations recorded in mm (1 in. = 25.4 mm). The axes labels ST0 through ST5 refer to the applied stress states, see Table 2.

The LVDT measurements were used to calibrate the filtering and scaling parameters for the Top sensors in both specimens (Top-CO, and Top-GG) as they measured the displacement throughout the entire length of specimen, which provides a measure of the specimen’s overall stiffness. After calibrating, the same parameters were applied to the Mid sensor (Mid-CO, and Mid-GG). Even with error minimization and data filtering, some noise due to inherent sensor uncertainty could not be filtered out. However, this uncertainty is consistent across all measurements. As a result, the algorithm predictions should be interpreted with a primary focus on comparison, especially between Mid and Top sensors, instead of the absolute values. Position z-coordinates were computed for each stress state for the top and middle sensors in both control and geogrid specimens. The resilient displacements were then obtained as the absolute difference between the z-coordinates corresponding to the peak load of the repeated load pulse. The resulting resilient deformations are presented in Figure 7a,b.

As evident from the results, with an appropriate scaling factor and parameter tuning, the Top sensor measurements can be reasonably matched with the LVDT measurements on the bulk specimen. The optimized algorithm then produces reasonable estimates of the Mid sensor position. Moreover, as tabulated in Table 4, the movement reduction ratios of resilient deformation measured by the Top sensor and the Mid sensor for the control and the geogrid specimens are 1.05 and 1.26, respectively. The ratios represent the reduction in an aggregate particle movement placed in the middle of the triaxial specimen compared to the top of the specimen. Due to aggregate–geogrid interlock near geogrid, it is expected that the resilient deformations will be smaller near the geogrid when compared to the top of the specimen. The results highlight the position tracking ability of the Smart Pebble sensors when paired with the position tracking algorithm. Additionally, it provides evidence that, with the proposed algorithm, the aggregate–geogrid interlock can be effectively quantified under the pulsed loading.

### 5.3. Shear Wave Velocity

**Figure 8:**
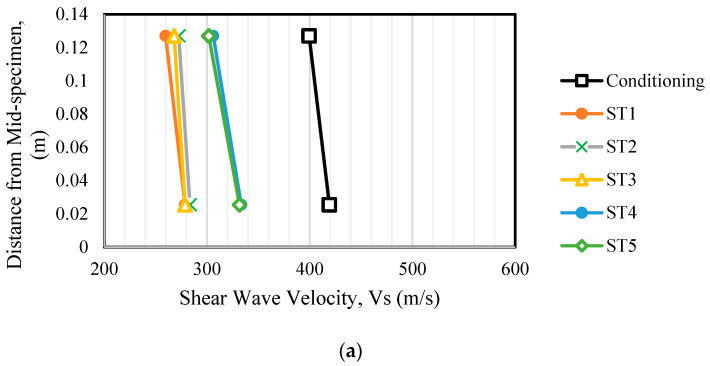

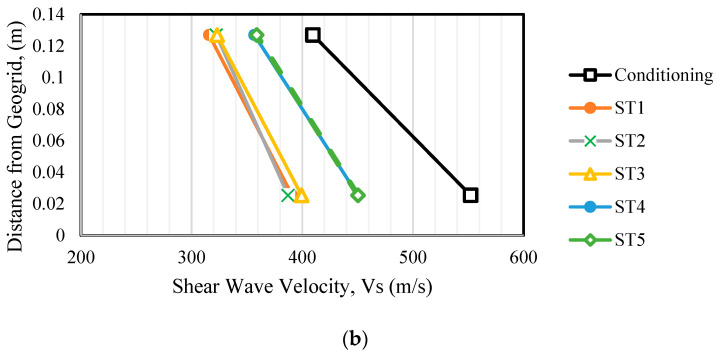
Shear wave velocity profiles for (**a**) control and (**b**) geogrid specimens.

Shear wave velocity profiles of the control and geogrid test specimens were measured using a set of two BE sensor pairs and are presented in Figure 8a,b. Owing to the highest confinement and the highest deviatoric stress, $V_{s}$ was observed to be highest for the conditioning stage. A typical trend of increasing $V_{s}$ was observed among the stress states ST1 through ST5. Additionally, the $V_{S}$ profiles are closely grouped based on the confinement applied for each stress state. These observations confirm the findings of previous researchers [6]. The $V_{s}$ ratios between the top and bottom BE sensors were observed as 1.06 and 1.257 for control and geogrid specimens, respectively. Higher shear wave velocity measurements near the geogrid (specimen mid-height) location, when compared to the top of the specimen location (see Figure 8b), clearly indicate the local stiffness enhancement brought by geogrid. For the control test, the top and middle $V_{s}\;$ measurements are quite similar, which agrees with Smart Pebble measurements.

## 6. Conclusions

This paper introduced Smart Pebble sensing technology and its accompanying aggregate particle tracking algorithm and sensor arrangements which have been recently developed to quantify inter-particle responses when installed in constructed assemblies of geomaterials subjected to repeatedly applied vehicular traffic loads. The algorithm for data processing and movement tracking encapsulates scaling and parameter tuning within a Kalman filter recursive feedback loop. Triaxial test specimens were prepared using a dense-graded aggregate material with two of the specimens also stabilized with a biaxial geogrid placed at specimen mid-height. Two Smart Pebble sensors were strategically positioned near the geogrid and towards the top of the triaxial specimen. The study also employed the proven technology of bender element (BE) sensors to corroborate the efficacy of the geogrid stabilization measured using the Smart Pebble sensors.

The following key conclusions can be drawn from the results of this investigation. Firstly, through the application of a Kalman filter-based algorithm and scaling procedure, the Smart Pebbles successfully captured the microscale resilient movements within a triaxial specimen. Secondly, once attuned to a specific physical state, the position tracking algorithm requires no additional parameter tuning and is equipped to manage uncertain signal noise, underscoring the potential for future field application of the Smart Pebble. Lastly, the ratio of resilient deformations measured at the top of the specimen and just above the geogrid was calculated to be 1.27. The BE sensors corroborated this result as the shear wave velocity ($V_{s}$) measured near the geogrid was nearly 1.26 times than that measured near the top of the specimen. These findings greatly demonstrate the successful preliminary applications of the Smart Pebble sensor and Kalman filter-based position tracking algorithm in quantifying the resilient response improvement of aggregates due to a geogrid under repeated loading. This further suggests that the use of Smart Pebbles within a laboratory or field context can aid engineers and designers in making informed decisions to ensure designs of mechanically stabilized pavement foundation layers.

The current study introduced a substantial initial understanding into the potential applications of Smart Pebble sensing units for continuous and precise assessment of the thickness designs and field performance trends of road foundation layers. However, a limitation of the system is the wired data extraction; future enhancements to the sensor should comprise a wireless communications unit and a superior battery for remote operation. With such wireless capabilities, an in situ assessment of the sensors and the position tracking algorithm is possible. Full scale testing could be undertaken as a future step for field implementation and could be paired with repeated load testing using devices such as the automated plate load testing (APLT) equipment. Future research should also focus on confirming the compatibility of the position tracking algorithm with other inertial measurement unit (IMU) sensors. Importantly, due to variances in sensor specifications, the existing algorithm may not be universally applicable to all IMU sensors available in the market. To address this limitation, subsequent research could explore the use of supervised machine learning tools on extensive datasets gathered through a variety of sensing units.

## Figures and Tables

**Table 1:** Location and data acquisition details of the Smart Pebble sensors.

Sensor ID	Geogrid	Z Coordinate from the Top of Specimen, in. (mm)	Sampling Frequency, Hz	Low Pass Filter, Hz
Top-CO	N	1 (25)	208	136
Mid-CO	N	5.5 (140)	104	
Top-GG	Y	1 (25)	416	
Mid-GG	Y	5.5 (140)	416	

Note: CO refers to the control specimen and GG refers to the geogrid-reinforced specimen.

**Table 2:** Applied stress states.

Stress State (ST)	Confining Stress, S_3_		Max. Axial Stress, S_max_		Cyclic Stress, S_cyclic_		Constant Stress, 0.1S_max_		No. of Load Applications
	kPa	psi	kPa	psi	kPa	psi	kPa	psi	
ST0	103.4	15	103.4	15	93.1	13.5	10.34	1.5	1000
ST1	20.7	3	20.7	3	18.6	2.8	2.07	0.3	100
ST2	20.7	3	41.4	6	37.3	5.4	4.14	0.6	100
ST3	20.7	3	62.1	9	55.9	8.1	6.21	0.9	100
ST4	34.5	5	34.5	5	31.0	4.5	3.45	0.5	100
ST5	34.5	5	68.9	10	62.0	9.0	6.89	1.0	100

**Table 3:** Geogrid specifications.

Property	Value
Nominal Aperture Dimensions	1.3 in. (33 mm)
Minimum Rib Thickness	0.03 in. (0.76 mm)
Number of Apertures in the Specimen	8 nos.
Polymer Material	Polypropylene

**Table 4:** Computed resilient deformations.

Stress Stage	Resilient Deformation (mm)							
	Control Specimen				Geogrid Specimen			
	LVDT	Top-CO (208 Hz)	Mid-CO (104 Hz)	Top-CO/Mid-CO	LVDT	Top-GG (416 Hz)	Mid-GG (416 Hz)	Top-GG/Mid-GG
ST0	0.198	0.148	0.124	1.194	0.203	0.157	0.140	1.121
ST1	0.032	0.032	0.032	1.016	0.030	0.033	0.022	1.500
ST2	0.044	0.053	0.052	1.019	0.057	0.064	0.055	1.164
ST3	0.070	0.087	0.075	1.168	0.089	0.120	0.092	1.304
ST4	0.036	0.044	0.047	0.946	0.047	0.054	0.041	1.317
ST5	0.065	0.082	0.085	0.959	0.084	0.120	0.101	1.188
			Average	1.050			Average	1.266
			Std. Dev.	0.096			Std. Dev.	0.127
			COV (%)	9.2			COV (%)	10.0

Note: 1 in. = 25.4 mm.

## Data Availability

The data presented in this study are available on request from the corresponding author.
